# More Limitations to Monolingualism: Bilinguals Outperform Monolinguals in Implicit Word Learning

**DOI:** 10.3389/fpsyg.2016.01218

**Published:** 2016-08-15

**Authors:** Paola Escudero, Karen E. Mulak, Charlene S. L. Fu, Leher Singh

**Affiliations:** ^1^The MARCS Institute for Brain, Behaviour and Development, Western Sydney University, PenrithNSW, Australia; ^2^Centre of Excellence for the Dynamics of Language, Australian Research Council, CanberraACT, Australia; ^3^Department of Psychology, National University of SingaporeSingapore, Singapore

**Keywords:** monolinguals, simultaneous bilinguals, implicit word learning, minimal pairs, phonetic detail, bilingual advantage

## Abstract

To succeed at cross-situational word learning, learners must infer word-object mappings by attending to the statistical co-occurrences of novel objects and labels across multiple encounters. While past studies have investigated this as a learning mechanism for infants and monolingual adults, bilinguals’ cross-situational word learning abilities have yet to be tested. Here, we compared monolinguals’ and bilinguals’ performance on a cross-situational word learning paradigm that featured phonologically distinct word pairs (e.g., BON-DEET) and phonologically similar word pairs that varied by a single consonant or vowel segment (e.g., BON-TON, DEET-DIT, respectively). Both groups learned the novel word-referent mappings, providing evidence that cross-situational word learning is a learning strategy also available to bilingual adults. Furthermore, bilinguals were overall more accurate than monolinguals. This supports that bilingualism fosters a wide range of cognitive advantages that may benefit implicit word learning. Additionally, response patterns to the different trial types revealed a relative difficulty for vowel minimal pairs than consonant minimal pairs, replicating the pattern found in monolinguals by Escudero et al. (2016) in a different English accent. Specifically, all participants failed to learn vowel contrasts differentiated by vowel height. We discuss evidence for this bilingual advantage as a language-specific or general advantage.

## Introduction

Typically, a person has learned 10s of 1000s of words by adulthood. While many of these words are learned explicitly, through instruction or clear, coinciding presentation of the word and its referent, not all words are learned in this manner. Some words are learned implicitly, by tracking the occurrence of an auditory word across multiple presentations in the context of multiple candidate referents. Humans are powerful statistical learners, and through this ability can implicitly derive the most likely referent of a novel word based on the likelihood of a candidate referent occurring simultaneously with an auditory word.

This type of learning, commonly termed *cross-situational word learning* (XSWL), appears staggering when one considers that the world presents learners with a seemingly infinite number of candidate referents for a single word in any one moment in time (Quine, 1960). Nonetheless, evidence shows that both infants (Smith and Yu, 2008; Vouloumanos and Werker, 2009; Vlach and Johnson, 2013) and adults (Yu and Smith, 2007; Smith et al., 2011; Suanda and Namy, 2012; Yurovsky et al., 2013; Dautriche and Chemla, 2014) can learn novel words through XSWL.

In a typical XSWL experiment, participants are presented with a series of ambiguous learning trials consisting of multiple objects and multiple words, with no explicit indication of word-object correspondences. During the learning phase, participants are not given instruction with regard to the nature of the task, and instead are simply asked to view the trials. After the learning phase, participants are presented with a forced-choice test in which they are asked to identify object-label mappings.

Studies on XSWL have typically included words that contained gross phonological differences (e.g., BLICKET vs. GAX; Smith and Yu, 2008; Vlach and Johnson, 2013). For pairs like this, listeners do not need to pay attention to fine phonological detail to differentiate competitor words and therefore do not need to pay attention to such information to allow learning. However, real-world word learning requires that words be encoded with fine phonological detail due to the presence of many phonologically overlapping words. The most extreme case of phonological overlap is seen in minimal pairs, in which words differ by only a single segment (e.g., TIP-DIP or TIP-TAP).

Recently, Escudero et al. (2016) asked whether adults in Sydney, Australia could learn novel words produced in Australian English via XSWL while simultaneously encoding fine phonological detail. In their experiment, participants viewed two side-by-side novel images during training, and heard the novel name associated with each image, without indication as to whether the words were named left-to-right or right-to-left. The words comprised eight CVC words in which four words differed by only one consonant (BON, DON, PON, TON), and the other four differed by one vowel (DEET, DIT, DOOT, DUT). During the test, in each trial the named image was paired with a distractor image. Based on the word associated with each image, this target-distractor pair formed either a non-minimal pair, in which two or all three segments differed (e.g., BON-DEET, DON-DEET), a consonant minimal pair, in which the initial consonant differed (e.g., BON-DON), or a vowel minimal pair, in which the vowel differed (e.g., DEET-DIT). Escudero et al. (2016) found that adults were able to learn all pair types via XSWL, but that performance was weakest in the context of a vowel minimal pair, indicating that phonological encoding of vowels was weaker than encoding of consonants.

Like monolinguals, bilinguals most certainly can and do learn words via cross-situational learning. However, it is unclear whether or how exposure or mastery of more than one language affects their learning relative to monolinguals. Bilingualism is often associated with greater performance on tests of executive function, selective attention and inhibitory control (e.g., Bialystok et al., 2006; Carlson and Meltzoff, 2008; Bialystok and Viswanathan, 2009; Bialystok and Craik, 2010). For instance, in the Stroop task, the names of colors are presented on a screen, and the color of the text either matches or mismatches the written color. Participants are then asked to name the color of the text, rather than the read the written word. Compared to monolinguals, bilinguals named the color of the text more quickly when the color of the text did not match the written color (Bialystok et al., 2008).

Bilingual advantages have been found in the linguistic domain as well. Using an explicit novel word learning paradigm, Kaushanskaya and Marian (2009) taught monolingual English speakers and early Spanish–English and Mandarin–English bilinguals 48 novel auditory words constructed from an artificial phonological system unfamiliar to all groups. After hearing each word, participants were shown its English orthographic translation. During the test phase, participants heard one of the novel words and were asked to select its English orthographic translation from five options. Both the English–Spanish and English–Mandarin bilinguals outperformed the English monolinguals when tested immediately after the learning phase, and 1 week later. In a follow-up study, Spanish–English bilinguals also outperformed English monolinguals when the words were comprised of phonemes that occurred in both English and Spanish (Kaushanskaya, 2012).

Kaushanskaya (2012) proposed that bilinguals’ advantage in novel word learning may be due to an enhanced phonological short term memory. Indeed, this proposal corresponds to research demonstrating that bilingualism confers gains in phonological working memory (Service et al., 2002; Majerus et al., 2008; Adesope et al., 2010), and also to research showing that multilinguals demonstrate better performance in digit-span and non-word repetition tasks (Papagno and Vallar, 1992). To test this proposal, Kaushanskaya (2012) divided monolinguals into high- and low-span phonological memory groups and tested them alongside bilinguals in their learning of novel phonologically familiar and unfamiliar words. Bilinguals outperformed both groups of monolinguals, suggesting that the bilingual advantage on this task may not be sufficiently explained by differences in phonological memory span.

But at a conceptual level, bilingualism might be expected to result in poorer or slower performance in some language abilities relative to monolinguals due to increased competition. During the course of spoken word recognition, competitor words are activated. For instance, the word CAT is activated during perception of the word CATALOG (e.g., Norris and McQueen, 2008). Because bilinguals have a lexicon in each language, there are more potential words that could be activated in the bilingual lexicon relative to monolinguals. Spoken word recognition is more difficult with increasing activation of competitors (Luce and Pisoni, 1998), and in the same way, the enlarged lexical space of bilinguals could be expected to interfere more with novel word learning. However, as described above, bilinguals typically show equal or enhanced word learning relative to monolinguals, possibly suggesting that they are able to suppress competitor activation in the non-target language. The general advantages in executive control discussed above may emerge from the need to control access and parallel activation between the bilinguals’ two languages, which takes place through enhanced attention to one language and/or inhibition of the other (e.g., Bialystok and DePape, 2009; Costa et al., 2009; Festman et al., 2010; Blumenfeld and Marian, 2011; Kroll and Bialystok, 2013; Duncan et al., 2016). Indeed, the areas of the brain involved in domain general executive control significantly overlap with the areas used in language control in bilinguals (Bialystok et al., 2012; Pliatsikas and Luk, 2016).

Experimental support for the suggestion that the executive control advantages commonly found in bilinguals are linked to their negotiation of access to their two languages comes from a word learning experiment in which Spanish–English bilinguals and English monolinguals learned novel translations for pictures of known items. At test, participants heard a newly learned word while viewing a target and distractor item, and were asked to click on the corresponding item. In some trials, the familiar word associated with the distractor image overlapped phonologically with the target word (e.g., the new word SHUNDO was associated with a picture of an acorn, and during test the acorn image was paired with a picture of a shovel, which shares the same onset as SHUNDO). Although, bilinguals had more phonological competitor words compared to monolinguals through their knowledge of words in two languages rather than just one, bilinguals looked less to the competitor images than did monolinguals, and mouse-tracking results showed that they tracked more directly to the target image (Bartolotti and Marian, 2012).

While bilingual advantages have been demonstrated in explicit word learning tasks, it is not clear how bilingualism might affect *implicit* word learning. Bartolotti et al. (2011) compared monolingual and bilinguals’ ability to extract and learn novel words composed of pure tones based on Morse code by tracking transitional probabilities in a continuous auditory stream. Participants with high bilingual experience, defined as higher reported L2 proficiency, earlier age of L2 acquisition, and higher frequency of L2 use, were better at learning words through tracking transitional probabilities than those with low bilingual experience. Inhibitory control strength (as measured by the Simon task) did not affect performance. When the bilingual participants were subsequently exposed to a different Morse code auditory stream containing conflicting transitional probabilities compared to the first stream, strength of inhibitory control (but not bilingual experience) aided performance, presumably through participants’ ability to suppress the influence from the former Morse code “language.” The authors proposed that the contribution of bilingual experience was perhaps due to increased phonological working memory. Although, this does not appear to explain the bilingual advantage for explicit word learning, it may have more of an effect on implicit learning (Bartolotti et al., 2011). Alternatively, while effects of increased phonological memory and enhanced executive functioning do not reliably explain the bilingual advantage when compared to skill-matched monolingual peers, bilingualism may nonetheless support these cognitive skills such that they are stronger in the bilingual population as a whole compared to monolinguals (see Kaushanskaya, 2012).

Importantly, bilingual advantages are not always found. With regard to the ability to form pairings between stimuli – a skill inherent to cross-situational word learning – there have been instances of finding no bilingual advantages in learning of non-linguistic tone-symbol pairings (Blumenfeld and Adams, 2014) and novel word-abstract referent pairings (Kaushanskaya and Rechtzigel, 2012). As well, a review of the existing literature investigating bilingual advantages in enhanced executive control found inconsistent evidence of such an advantage (Hilchey and Klein, 2011), and this has been further supported through subsequent empirical research (Kousaie and Phillips, 2012a,b; Paap and Greenberg, 2013). Paap (2014) recently proposed that the generally accepted notion of a bilingual advantage, at least in executive functioning, may be the result of a publication bias. This is supported by a meta-analysis of subsequent publication rates of studies submitted as conference abstracts, based on whether their findings supported or challenged the notion of a bilingual advantage in executive functioning (de Bruin et al., 2015). Alarmingly, the analysis showed a clear publication bias. While the number of conference abstracts supporting and challenging the bilingual advantage in executive functioning were similar (54 vs. 50, respectively), 63% of the studies in support of the bilingual advantage went on to be published as full journal articles, compared to only 36% of the studies that challenged the bilingual advantage. Thus, bilingual advantages in executive functioning, and perhaps in other areas, are very likely not as pervasive, and are likely weaker, than has been generally understood.

One important factor that may influence whether a bilingual advantage is measured in the linguistic domain is the relationship between the linguistic stimuli and the listeners’ phonological space. Models such as PAM (Perceptual Assimilation Model; Best, 1994, 1995), its extension to non-native and second language (L2) learning (PAM-L2; Best and Tyler, 2007) and L2LP (Second Language Linguistic Perception model; Escudero, 2005, 2009; van Leussen and Escudero, 2015) say that perception of non-native contrasts that do not exist in a learner’s native language is generally expected to be worse than perception of non-native contrasts that have a counterpart in the learner’s native language (though both models claim that the relationship between native and non-native phones predicts perception of specific non-native contrasts). Thus, infants, children, and adults who learn two languages from birth may have more difficulty or fail to show an advantage if a contrast is absent in one or both of their languages. By extension of this proposal, research comparing novel word learning in monolingual and bilingual infants has shown that when bilinguals are familiar with phonological contrasts in both test languages, they outperform monolinguals (Mattock et al., 2010; Singh et al., 2016). However, other research has found that bilingual infants exposed to English and another language are delayed in novel word learning of minimal pairs relative to English monolinguals, and that this delay is independent of the similarity of the English phonological contrast being tested with the analogous contrast in their second language (Fennell et al., 2007). It remains an open question whether the phonological status of a contrast affects a possible bilingual advantage in adulthood, and whether factors such as language dominance and age of acquisition of the L2 correlate with any such effect.

In Escudero et al. (2016) examination of cross-situational word learning of minimal pair words, 40 of the 71 total participants reported proficiency in one or more languages in addition to English; however, no effect of bi/multilingualism was found. While this lack of a bilingual advantage may reflect a lack of a bilingual advantage in implicit word learning, the null result may instead stem from the heterogeneity of the bilingual sample with regard to several factors that may be related to cognitive advantages associated with bilingualism. For instance, age of acquisition of a second language (L2) has been shown to affect performance on the flanker task (Eriksen and Eriksen, 1974), which measures response inhibition. Early bilinguals who acquired their L2 before the age of 10 outperformed late bilinguals and monolinguals (Luk et al., 2011). As well, bilinguals who switch between their languages more frequently outperform those who switch less frequently in measures of executive control (e.g., Prior and Gollan, 2011; Verreyt et al., 2016).

To test whether a bilingual advantage occurs in implicit word learning, we compared performance by Australian English monolinguals from Sydney, Australia with a homogeneous population of Singaporean English–Mandarin simultaneous bilinguals from Singapore. We tested their XSWL of the same non-minimal and minimal pair words used by Escudero et al. (2016), but produced by an American English speaker, so that the accent would not be native to either group, but would be familiar to both groups (e.g., through media). Thus, in line with research demonstrating a bilingual advantage in explicit word learning (Kaushanskaya and Marian, 2009; Kaushanskaya, 2012), and based on our supposition that XSWL would be aided by executive functioning features, for which bilinguals have often been found to have an advantage over monolinguals (e.g., Adesope et al., 2010; Kroll and Bialystok, 2013), it was predicted that our bilingual participants would outperform our monolingual participants when learning novel word pairs in an implicit learning paradigm, at least when tested in a non-minimal pair context. Secondly, we predicted that accuracy by both groups would be poorest for vowel minimal pair trials, which would replicate the finding by Escudero et al. (2016). Lastly, all words in the present study were comprised of phonemes present in (or analogous to) Australian English (Cox and Palethorpe, 2007), Singaporean English (Wee, 2004, 2010; Deterding, 2007) and Standard Mandarin (Duanmu, 2002; which is phonologically similar to Singaporean Mandarin), with the exception that the vowels /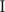 and /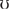/, found in the novel words DIT and DUT are not present in Singaporean English or Standard Mandarin. The L2LP model (Escudero, 2005; van Leussen and Escudero, 2015) predicts that non-native vowels may be perceived as acoustically proximate native vowels. While Mandarin does contain the vowel /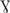/, which is acoustically proximate to /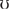/, the most acoustically proximate vowel to /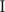/ is /i/, as in DEET, which may lead to confusion in learning and discriminating DIT-DEET, which is differentiated by vowel height. Thus, we predicted that our Singaporean bilinguals would show poorer performance for vowel contrasts differentiated by height relative to monolinguals.

## Materials and Methods

### Participants

All participants were native English speakers at English-language universities. Monolingual participants were 16 monolingual Australian English (henceforth, AusE) speakers aged 17.1–37.0 years (*M*_age_ = 24.3, *SD* = 5.9, 10 females) who were primarily undergraduate students at Western Sydney University. These participants received course credit or $10 travel compensation for their participation. Bilingual participants were 15 simultaneous Singaporean English–Mandarin (henceforth, SE–SM) bilinguals aged 20.0–23.5 years who were undergraduate students from the National University of Singapore (*M*_age_ = 21.6, *SD* = 1.1, 9 females). These participants received $5 SGD compensation for participation. None of the participants reported a history of hearing or language impairment. Participants’ language background was determined via a language background questionnaire administered at the beginning of the session. Participants were determined to be AusE monolinguals if all parents or caretakers were born in Australia and were native speakers of AusE, and if the participant reported that during childhood they did not regularly spend time with someone whose native language was not AusE (e.g., a close relative or family friend, and/or someone who lived with them). Participants were determined to be SE–SM simultaneous bilinguals if they received exposure to both SE and SM by 2 years of age, and reported current proficiency in both SE and SM. When asked to rate their oral comprehension and productive proficiency on a seven-point Likert scale (7 = native), monolinguals’ average rating for their English comprehension ability was 7.0 (*SD* = 0.0), and was 6.9 (*SD* = 0.2) for their productive ability. On average, bilinguals reported their English comprehension ability as 6.7 (*SD* = 0.7), and their production ability as 6.6 (*SD* = 0.7), and these values did not significantly differ from monolinguals’ ratings. Bilinguals rated their Mandarin comprehension ability as 5.8 (*SD* = 1.4), and their production ability as 5.1 (*SD* = 1.7). Participants gave informed consent prior to participation in accordance to the Western Sydney University Human Research and Ethics Committee and National Singapore University Institutional Review Board.

### Stimuli

#### Novel Words

**FIGURE 1:**
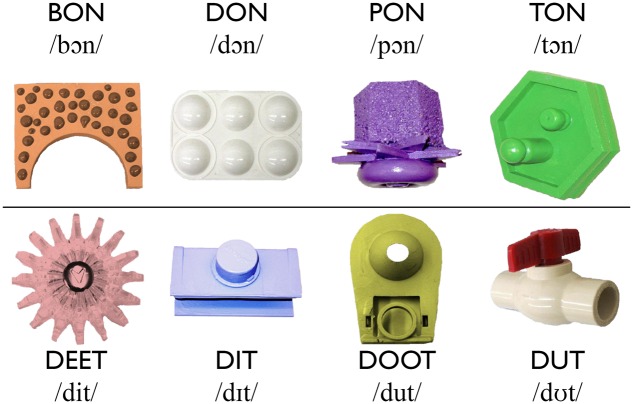
**The eight novel words and their visual referents.** The vowel used for the consonant minimal pairs in the top row is /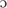/ as in POT. The vowels used in the vowel minimal pairs are /i/ as in BEAT, /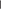/ as in BIT, /u/ as in BOOT, and /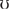/ as in BUT.

Eight monosyllabic nonsense words were recorded by a female native speaker of American English. As shown in **Figure 1**, the words followed a CVC structure, and adhered to English phonotactics. The words have been used in previous research on the acquisition of minimal pairs (Curtin et al., 2009; Fikkert, 2010), including in a cross-situational word learning context, which used the same set of words recorded by a native female speaker of AusE, produced with the same intonation contours as the present study (Escudero et al., 2016, under review). Four of the words differed minimally in their first consonant, whereas the other four differed in their vowel. All words were comprised of phonemes present in (or analogous to) AusE (Cox and Palethorpe, 2007), SE (Deterding, 2007; Wee, 2010) and Standard Mandarin (Duanmu, 2002; which is phonologically similar to SM), with the exception that the vowels /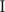/ and /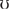/ found in the novel words DIT and DUT are not present in SE or Mandarin, though Mandarin does contain the vowel /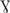/, which is acoustically proximate to /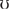/. Two tokens of each of the eight spoken words were selected for use in the experiment so that intonation contours were comparable across words.

#### Novel Visual Referents

The visual referents for the words were pictures of novel items used in previous studies on XSWL (Vlach and Sandhofer, 2014; Escudero et al., 2016, under review). Each nonsense word was randomly paired once with a visual referent (**Figure 1**). The same word-referent pairings were presented to all participants, and were the same pairs used in previous studies on cross-situational word learning (Escudero et al., 2016, under review). Each image measured 280 × 274 pixels. Slides were created in which two of the eight visual referents were placed on an 800 × 600-pixels white background with the top-left corner of the left images positioned at 20 × 163 pixels, and the top-left corner of the right image positioned at 500 × 163 pixels.

#### Attention Videos

Each attention video consisted of a looped cartoon animation measuring 170 × 170 pixels, which was centered on the monitor between every third trial in the learning phase and between each trial in the testing phase. Each animation was paired with a non-linguistic sound.

### Procedure

**FIGURE 2:**
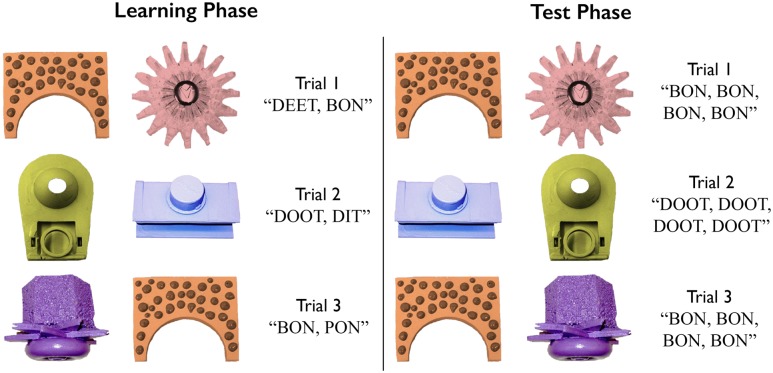
**Examples of learning and test trials**.

The procedure was identical to that reported in Escudero et al. (2016), and consisted of a learning phase and testing phase. Examples of learning and testing phase trials can be seen in **Figure 2.** At the beginning of the experiment, participants were seated in front of a 19-in. display and were told that they would watch some images on the screen and hear some words. Participants were not told that the words were names for the images, nor were they asked to try and discover which word was paired with which image.

#### Learning Phase

The learning phase consisted of 36 trials, across which participants were presented with each word-referent pairing nine times. In each learning trial, two of the eight visual referents were displayed on the screen. After 500 ms, the word corresponding to each item was spoken so that each picture was named once, either left-to-right, or right-to-left, with 500 ms between spoken words. There were no cues that signaled whether the visual referents were named left-to-right or right-to-left.

The presentation order of the paired trials was randomized for each participant and the pairings were controlled such that each visual referent occurred with every other visual referent at least once, and no more than twice. If the same pairing occurred more than once, the designations of the left and right image were swapped so that participants never saw the exact same visual pairing more than once. As each word appeared nine times, the occurrence of an image in the left or right position was balanced such that half of the words appeared five times on the left and four times on the right, while the other half appeared in the opposite pattern. Whether a visual referent was named first or second, and the number of times each of the two tokens of each nonsense word were heard, were also balanced.

The two words presented in each trial belonged to one of three possible phonological relationships when paired: non-minimal pairs (non-MPs) differed in two or all three segments (e.g., BON-DEET, DON-DEET); consonant minimal pairs (cMPs) differed in their initial consonant (BON-TON), and; vowel minimal pairs (vMPs) differed in their vowel (DEET-DIT). Further, cMPs differed either by place (BON-DON, PON-TON), voicing (BON-PON, DON-TON), or both place and voicing (DON-PON, TON-BON), and vMPs differed by height (DEET-DIT, DOOT-DUT), backness (DEET-DOOT, DUT-DIT), or both height and backness (DUT-DEET, DIT-DOOT). During training, participants were exposed to 24 non-MPs, and all 6 cMPs and 6 vMPs, for a total of 36 pairs. Each learning trial lasted 3.5 s and an attention getter comprising a 170 × 170 centrally presented looped video paired with a non-linguistic sound, played between every third trial until the participant’s gaze was centrally fixed. The total duration of the learning phase was approximately 3 min. Examples of training trials are presented in **Figure 2.**

#### Test Phase

After completion of the learning phase, participants were seated in front of a laptop computer with a 15-in. monitor. Participants were instructed that they would see two images on the screen from the same set of images they had just watched. They were told they would hear the name corresponding to one of the images, and should indicate by pressing the left or right ALT key whether they believed the word corresponded to the image on the left or right, respectively. Test trials contained the same pairs of two words and visual referents as the learning phase, but the left and right designations of the images were randomized once such that for half of the trials, the order of the images was swapped relative to the training phase. Each participant received the same test trials, presented in three counterbalanced blocks of 12, with the trials within each block occurring in a random order. For each trial, once the two images had been on the screen for 500 ms, two tokens of the spoken word corresponding to one of the images (the target object) played twice each in an alternating fashion with 500 ms between each repetition, such that the participant heard the word a total of four times. Each word served as the target four or five times. As in the training phase, the test consisted of 24 non-MP trials, 6 cMP trials, and 6 vMP trials. Each trial lasted 6.5 s, resulting in a test phase duration of approximately 4 min. Examples of test trials are presented in **Figure 2.**

## Results

**FIGURE 3:**
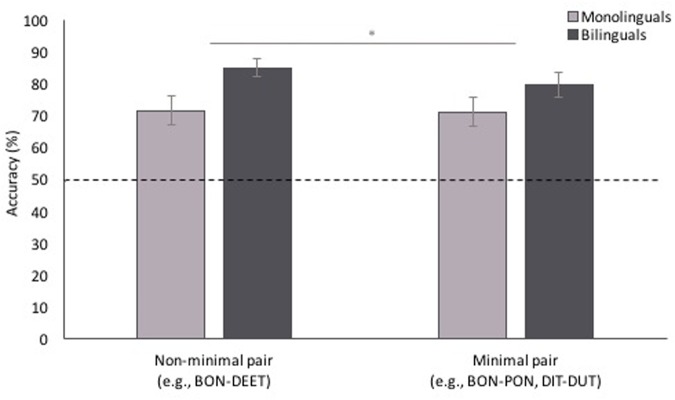
**Accuracy for non-minimal pair and minimal pair test trials.** Participants were more accurate for non-minimal pair than minimal pair trials, and bilingual participants were more accurate overall than monolingual participants. Error bars represent one standard error. ^∗^*p* < 0.05.

No-response trials, which comprised 1.3% of the total sample, were removed from analysis. To examine whether there were differences in word learning performance between non-MP and MP trials, and to compare bilinguals’ performance relative to monolinguals, participants’ correct and incorrect responses were analyzed in a mixed-effects binary logistic model with pair type (non-MP, MP) and language background (monolingual vs. bilingual) as fixed variables, and subject, order, target, distractor, and target location as random variables. A separate independent-samples *t*-test revealed a trend such that the monolingual group was marginally older than the bilingual group (*t*[16.17] = 1.76, *p* = 0.098, [-0.5, 5.8 years]). Thus, age was entered in the mixed-effects model as a random covariate. As can be seen in **Figure 3**, the model revealed a main effect of pair type [χ^2^(1, *n* = 1101) = 4.49, *p* = 0.034], with greater accuracy for non-MP than MP trials. There was also an effect of language background [χ^2^(1, *n* = 1101) = 5.02, *p* = 0.025]. Overall, bilinguals were more accurate than monolinguals. There was no interaction of language background and pair type [χ^2^(1, *n* = 1101) = 1.66, *p* = 0.198]. One-sample *t*-tests against chance showed that proportion fixation to the named image was above chance for both non-MP and MP trials, for both monolinguals (non-MP: *t*[15] = 4.67, *p* < 0.001, 95% CI [0.12, 0.32]; MP: *t*[15] = 4.52, *p* < 0.001, [0.11, 0.31]) and bilinguals (non-MP: *t*[14] = 12.49, *p* < 0.001, [0.29, 0.41]; MP: *t*[14] = 7.61, *p* < 0.001, [0.21, 0.38]). Thus, all learners were able to infer word-object pairings for both non-MP and MP trials.

**FIGURE 4:**
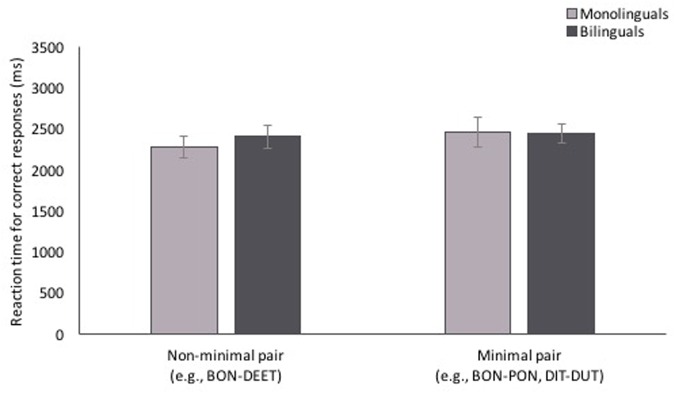
**Reaction time (RT) to non-minimal pair and minimal pair test trials.** RTs did not differ between pair types or language background. Error bars represent one standard error.

Participants’ reaction times (RTs) for correct responses, which are shown in **Figure 4**, were analyzed in a mixed-effects linear model with the same fixed and random factors and random covariate as in the accuracy analysis. There was no main effect of pair type [χ^2^(1, *n* = 857) = 2.40, *p* = 0.121], or language background [χ^2^(1, *n* = 857) = 1.24, *p* = 0.266], and no interaction between the two [χ^2^(1, *n* = 857) = 1.79, *p* = 0.181].

**FIGURE 5:**
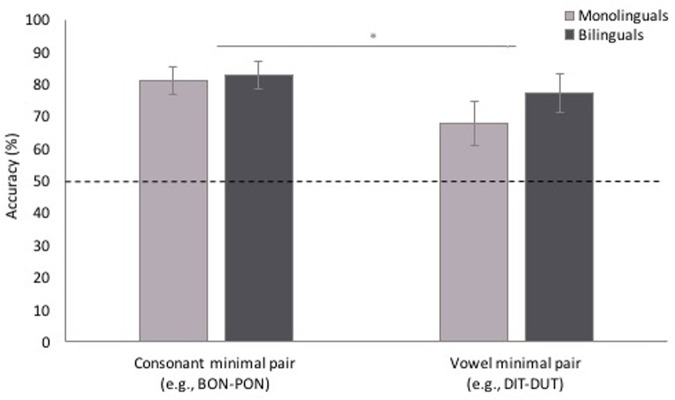
**Accuracy for consonant minimal pair and vowel minimal pair test trials.** Participants were less accurate for vowel minimal pairs than consonant minimal pairs. Error bars represent one standard error. ^∗^*p* < 0.05.

To answer our next question of whether performance differed depending on whether the MP differed in one consonant or one vowel, and whether participants’ language background affected performance, participants’ correct and incorrect responses for MP trials were analyzed in a mixed-effects binary logistic model with MP type (cMP, vMP) and language background (monolingual vs. bilingual) as fixed variables, and subject, order, target, distractor, and target location as random variables, and age entered as a random covariate. As shown in **Figure 5**, the model revealed a main effect of MP type [χ^2^(1, *n* = 368) = 5.01, *p* = 0.025], with greater accuracy for cMP than vMP trials. There was no effect of language background [χ^2^(1, *n* = 368) = 0.82, *p* = 0.366], and no interaction of language background and minimal pair type [χ^2^(1, *n* = 368) = 0.61, *p* = 0.435]. One-sample *t*-tests against chance showed that proportion fixation to the named image was above chance for both cMP and vMP trials, for both monolinguals (cMP: *t*[15] = 7.27, *p* < 0.001, [0.22, 0.40]; vMP: *t*[15] = 2.51, *p* = 0.024, [0.03, 0.33]) and bilinguals (cMP: *t*[14] = 7.89, *p* < 0.001, [0.24, 0.42]; vMP: *t*[14] = 4.65, *p* < 0.001, [0.15, 0.40]).

**FIGURE 6:**
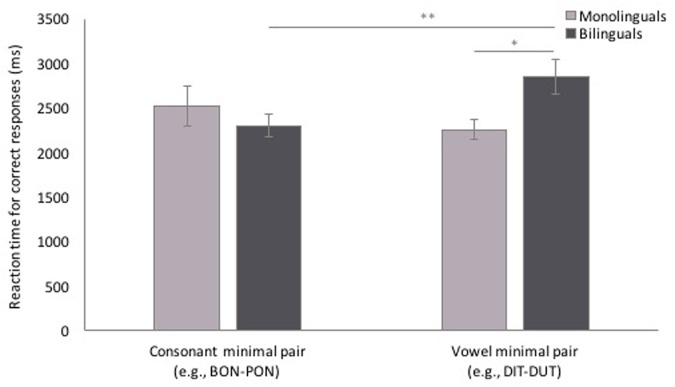
**Reaction time (RT) to consonant minimal pair and vowel minimal pair test trials.** Bilinguals had slower RTs for vowel minimal pair trials than consonant minimal pair trials, and had slower RTs to vowel minimal pair trials than monolinguals. Error bars represent one standard error. ^∗^*p* < 0.05, ^∗∗^*p* < 0.01.

Participants’ RTs for correct responses to MP trials were analyzed in a mixed-effects linear model with the same fixed and random factors and random covariate as in the minimal pair accuracy analysis. As seen in **Figure 6**, while there was no main effect of MP type [χ^2^(1, *n* = 284) = 1.35, *p* = 0.245], or language background [χ^2^(1, *n* = 284) = 1.19, *p* = 0.275], the interaction of MP type and language background was significant [χ^2^(1, *n* = 284) = 6.50, *p* = 0.011]. LSD-corrected pairwise comparisons showed that while monolinguals’ RT did not differ for cMP and vMP trials (*p* = 0.323, [-161.62 ms, 490.63 ms]), bilinguals were slower to respond to vMP trials than cMP trials (*p* = 0.009, [108.90 ms, 771.14 ms]). Further, while RT for cMP trials did not differ between monolinguals and bilinguals (*p* = 0.689, [-358.51 ms, 542.19 ms]), bilinguals were slower to respond to vMP trials than monolinguals (*p* = 0.022, [75.39 ms, 949.98 ms]).

**FIGURE 7:**
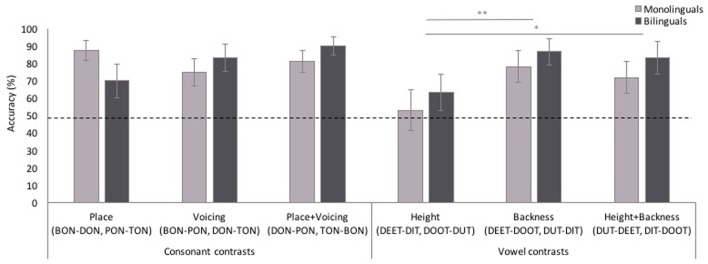
**Accuracy for consonant and vowel minimal pair trials by contrast type.** Performance did not differ across consonant contrasts, but participants were less accurate for vowel height contrasts compared to each other vowel contrast type. Error bars represent one standard error. ^∗^*p* < 0.05, ^∗∗^*p* < 0.01.

Finally, to determine whether participants’ performance for MP trials differed depending on the feature difference between the MPs, we analyzed participants’ correct and incorrect responses for cMPs and vMPs in two separate mixed-effects binary logistic models with contrast type (cMPs: place contrast, voicing contrast, place+voicing contrast; vMPs: height contrast, backness contrast, height+backness contrast) and language background as fixed effects, and subject, order, target, distractor, and target location as random factors, and age included as a random covariate. As can be seen in **Figure 7**, performance for cMPs did not differ depending on the contrast type [χ^2^(2, *n* = 183) = 1.14, *p* = 0.564], or language background [χ^2^(1, *n* = 183) = 0.13, *p* = 0.723], and there was no interaction of contrast type and language background [χ^2^(2, *n* = 183) = 3.57, *p* = 0.168]. However, for vMP trials, performance differed depending on the vowel contrast [χ^2^(2, *n* = 185) = 9.57, *p* = 0.008]. LSD-corrected pairwise comparisons showed that participants were less accurate for vowel contrasts differing in height only than for contrasts differing in both height and backness (*p* = 0.032, [-0.38, -0.02]) or backness only (*p* = 0.004, [-0.41, -0.08]). There were no effects of language background [χ^2^(1, *n* = 185) = 1.34, *p* = 0.247], nor was there an interaction of contrast type and language background [χ^2^(2, *n* = 185) = 0.08, *p* = 0.959]. One-sample *t*-tests against chance showed that both monolinguals and bilinguals demonstrated above chance performance for vowel contrasts differentiated by height+backness (monolinguals: *t*[15] = 2.41, *p* = 0.029, 95% CI [0.25, 0.41]; bilinguals: *t*[14] = 3.57, *p* = 0.003, [0.13, 0.53]) and backness only (monolinguals: *t*[15] = 3.09, *p* = 0.007, [0.09, 0.48]; bilinguals: *t*[14] = 4.79, *p* < 0.001, [0.20, 0.53]), but were both at chance for vowel contrasts differentiated by height only (monolinguals: *t*[15] = 0.27, *p* = 0.791, [-0.22, 0.28]; bilinguals: *t*[14] = 1.29, *p* = 0.217, [-0.09, 0.35]).

**FIGURE 8:**
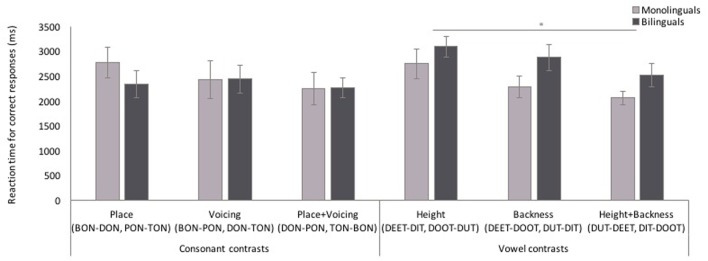
**Reaction time (RT) to consonant and vowel minimal pair trials by contrast type.** Performance did not differ across consonant contrasts. Participants had slower RTs to vowel height contrasts than height+backness contrasts, and bilingual participants had slower RTs to vowel minimal pair trials than monolinguals. Error bars represent one standard error. ^∗^*p* < 0.05.

Participants’ RTs for correct responses to cMP and vMP trials by contrast type (**Figure 8**) were analyzed in a mixed-effects linear model with the same fixed and random factors and random covariate as the accuracy analysis. RT did not differ based on the contrast type for the cMPs [χ^2^(2, *n* = 150) = 2.40, *p* = 0.301], and there was no effect of language background [χ^2^(1, *n* = 150) = 0.11, *p* = 0.742] or interaction with contrast type and language background [χ^2^(2, *n* = 150) = 1.21, *p* = 0.547]. However, for vMPs, RT did differ based on the contrast type [χ^2^(2, *n* = 134) = 6.60, *p* = 0.037], and language background of the participant [χ^2^(1, *n* = 134) = 5.21, *p* = 0.022], but there was no interaction between the two [χ^2^(2, *n* = 185) = 0.193, *p* = 0.908]. Overall, bilinguals were slower to respond to vMPs than monolinguals, and participants were slower to respond to contrasts differing in height only than contrasts differing on both height and backness (*p* = 0.013, [123.60 ms, 1072.24 ms]).

## Discussion

In this study, we compared Australian English monolinguals and simultaneous Singaporean English–Mandarin bilinguals in their learning of phonologically overlapping novel words in an implicit, cross-situational paradigm, comparing vowel and consonant minimal pairs and non-minimal pairs produced in American English. Participants from both groups were significantly above chance in their recognition of new words across all pair types, consistent with successful learning within this paradigm by adult listeners. While not unexpected, this result reinforces that this mechanism of word learning at least remains available to adult listeners, and also reassures as to the validity of the experimental paradigm.

Bilinguals outperformed monolinguals overall, which was consistent with our hypothesis, and also consistent with the interpretation that bilinguals have increased abilities in language-based tasks, hypothesized to be through enhanced phonological working memory (e.g., Kaushanskaya, 2012) and/or enhanced executive functioning skills (e.g., Bartolotti and Marian, 2012). In cross-situational word learning of phonologically overlapping words, enhanced phonological memory may allow for better implicit tracking of word-referent co-occurrence probabilities across trials, and augmented inhibitory control may allow for reduced activation of phonological neighbors in the lexicon.

Crucially, bilinguals’ greater accuracy relative to monolinguals in our experiment is unlikely to be due to increased exposure to or familiarity with the American English target accent, as it is likely that there is more exposure to American English in Australia than in Singapore. For instance, American television programs account for 26.94% of free-to-air television broadcast hours in Australia, comprising 694 of 2576 weekly broadcast hours across 23 channels (Australian Tv guide for free-to-air television, 2016), whereas American programming accounts for only 10.08% of free-to-air broadcast hours in Singapore, comprising 79 of 784 h weekly across 7 MediaCorp TV channels (Toggle Tv Guide, 2016).

Although, we did not measure socioeconomic status (SES) of participants in this experiment, we believe that is it unlikely that there were differences between our participant groups that could account for the pattern of results found. A difference in SES between groups has been shown to lead to differences in performance in the Simon task (a measure of inhibitory control strength), such that children from higher SES families performed better on the task compared to children from lower SES families (Morton and Harper, 2007). The authors reasoned that because bilingual families are typically of lower SES than monolingual families, studies that have not controlled for SES may be conflating SES with bilingualism. While this is more likely to be the case in predominantly monolingual communities, both of our participant groups represented the predominant homogeneous language group of their community. As well, both of our participant groups comprised students at major urban public universities in developed countries with lifelong educational instruction in English. Tuition fees for undergraduate psychology degrees (the degree undertaken by the majority of participants) at each university are comparable^1^, and both universities offer subsidized fees for nationals.

Moving to linguistic, rather than general cognitive aspects of this research, participants struggled more for minimal pair trials relative to non-minimal pairs, and in vowel minimal pair trials relative to consonant minimal pairs. Lower accuracy for vowel contrasts has previously been shown in native Australian English listeners’ cross-situational learning of minimal pair words in Australian English (Escudero et al., 2016), and the present study extends this finding to other varieties of Modern English. There are several factors that may contribute to this. Firstly, while consonants tend to be perceived categorically (Liberman et al., 1967), vowels are perceived in a more continuous manner in many languages, including English (Fry et al., 1962; Stevens et al., 1969; Beddor and Strange, 1982; Polka, 1995). This may make it more difficult to perceive differences between vowel minimal pairs relative to word pairs that contain consonant differences. In English, vowels are also proposed to play less of a lexical role than consonants in speech perception, and instead play more of a role in conveying suprasegmental and syntactic information to the perceiver (Nespor et al., 2003). Supporting this, research typically finds a perceptual bias toward consonants in tasks involving lexical access and processing (e.g., Cutler et al., 2000; Bonatti et al., 2005; Toro et al., 2008), including in explicit word learning by adults (Havy et al., 2014). Specifically, both monolinguals and bilinguals failed to discriminate vowel contrasts differing by height only, and also displayed slower RTs for these contrasts. Bilinguals were expected to have difficulty in discriminating the height contrast DEET and DIT due to the lack of the vowel in DIT in their phonological space. While it is not clear at this point whether a different factor accounted for monolinguals’ failure, or whether failure by both groups was due to an unforeseen factor is at this point unclear. Interestingly, Escudero et al. (2014) found that Australian English-learning infants could not discriminate an Australian English vowel height contrast embedded in a minimal pair in an explicit word learning task, perhaps suggesting that vowel height may be a more difficult cue to perceive through the lifespan. Ongoing research in our laboratory comparing Australian English monolinguals, Singaporean English–Mandarin simultaneous bilinguals, and Mandarin–Australian English late sequential bilinguals in their cross-situational learning of minimal pairs produced in Australian English and American English will further address this question.

Although, bilinguals did not demonstrate an overall difference in accuracy for minimal pair types compared to monolinguals, they were slower to respond to vowel minimal pair trials compared to monolinguals. As mentioned above, this may have been due to difficulty in perceiving the vowel /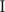/ in DIT, and in particular, discriminating it from DEET (/i/). As DEET and DIT were involved separately in all vowel contrast types, this may have led to bilinguals’ overall slower RTs for vowel minimal pair trials relative to monolinguals. Alternatively, this difference in performance may be due to differences between English and Mandarin. While experiments in English typically find a consonant bias, there is evidence that in Mandarin, vowels may contribute more to lexical identity than consonants. For instance, Chen et al. (2015) found that native Mandarin listeners showed better identification for Mandarin words made up of a consonant and vowel (CV words) when the consonant was replaced with noise (V-only words) than when the vowel was replaced with noise (C-only words). They also found that adding a proportion of the vowel aided identification of C-only words, while adding a proportion of the consonant to V-only words did not aid their identification, perhaps due to the fact that tone information is coupled with vowel information. It is therefore possible that apart from the consonant minimal pairs, in which every vowel was the same, for vowel minimal pairs, bilingual participants may have waited longer to respond in order to process the vowel information that is more lexically important to them compared to the monolinguals. Notably, this interpretation of the finding implies cross-talk between both of the bilinguals’ languages. Future work could test this interpretation by comparing bilinguals’ performance here with bilinguals whose languages both demonstrate a consonant bias rather than a vowel bias in word identification, such as English and French. Another possibility is that the same bias for consonants over vowels does not exist in native speakers of Singaporean English, which may be due to the local influence of Mandarin.

## Conclusion

Our study is the first to demonstrate that bilinguals can also learn words via cross-situational statistical learning, and can do so while encoding fine phonological detail. Both Singaporean English–Mandarin simultaneous bilinguals and Australian English monolinguals learned phonologically overlapping word-object pairings sufficiently as to identify visual referents corresponding to words spoken in American English in the context of minimal, as well as non-minimal pairs. Thus, the finding also generally replicates Escudero et al. (2016), who found that a separate set of Australian English speakers than those tested here could learn minimal pair words produced in their accent. More importantly, although research on explicit word learning has often found a bilingual advantage relative to monolinguals (e.g., Kaushanskaya and Marian, 2009; Bartolotti and Marian, 2012; Kaushanskaya, 2012), our findings demonstrate for the first time that bilinguals also outperform monolinguals in a cross-situational word learning task. Future research can now explore whether this bilingual advantage in word learning accuracy lies in general cognitive attributes such as increased verbal working memory or attention, or cultural factors (e.g., Yang et al., 2011). Alternatively, the advantage here may be specific to the linguistic background of the bilinguals (i.e., simultaneous English–Mandarin) and test language used (American English). Ongoing work in our lab will begin to address this latter issue by measuring performance in this task by late sequential Mandarin-English bilinguals and by bilinguals with different linguistic backgrounds, as well as in the same task but using a different English accent as the stimulus.

## Author Contributions

PE conceived the project and discussed bilingual testing with LS. KM programmed and set up the experiment in Sydney and sent instructions as well as checked for comparability of set up and results with CF, who implemented the set up in Singapore. CF collected data in Singapore. KM completed analyses. All authors wrote the paper.

## Conflict of Interest Statement

The authors declare that the research was conducted in the absence of any commercial or financial relationships that could be construed as a potential conflict of interest.

## Footnotes

1 For instance, many participants were undertaking a Bachelor of Arts degree. The annual cost of this degree at the National University of Singapore for 2016/2017 was $29,350 SGD (Fees for Undergraduate Programmes, 2016), and was $22,000 AUD for the 2016 academic year at Western Sydney University (Fees and Costs, 2016).
